# TSG-6 released from intraperitoneally injected canine adipose tissue-derived mesenchymal stem cells ameliorate inflammatory bowel disease by inducing M2 macrophage switch in mice

**DOI:** 10.1186/s13287-018-0841-1

**Published:** 2018-04-06

**Authors:** Woo-Jin Song, Qiang Li, Min-Ok Ryu, Jin-Ok Ahn, Dong Ha Bhang, Yun Chan Jung, Hwa-Young Youn

**Affiliations:** 1 Department of Veterinary Internal Medicine, College of Veterinary Medicine, Seoul National University, Seoul, 08826 Republic of Korea; 2 Department of Molecular and Cellular Biology, Samsung Biomedical Research Institute, Sungkyunkwan University School of Medicine, Suwon, Gyeonggi 16419 Republic of Korea; 3 KPC Corporation, Gwangju, Gyeonggi 12773 Republic of Korea

**Keywords:** TSG-6, Mesenchymal stem cells, Inflammatory bowel disease, Canine, Cell therapy, Immunomodulation, M2 macrophage

## Abstract

**Background:**

Inflammatory bowel disease (IBD) is an intractable autoimmune disorder that markedly deteriorates one’s quality of life. Mesenchymal stem cells (MSCs) alleviate inflammation by modulating inflammatory cytokines in inflamed tissues, and have been suggested as a promising alternative for IBD treatment in human and veterinary cases. Furthermore, tumor necrosis factor-α-induced gene/protein 6 (TSG-6) is a key factor influencing MSC immunomodulatory properties; however, the precise mechanism of TSG-6 release from canine MSCs in IBD remains unclear. This study aimed to assess the therapeutic effects of canine adipose tissue-derived (cAT)-MSC-produced TSG-6 in an IBD mouse model and to explore the mechanisms underlying the immunomodulatory properties.

**Methods:**

Mice with dextran sulfate sodium-induced colitis were administered cAT-MSCs intraperitoneally; colon tissues were collected on day 10 for histopathological, quantitative real-time polymerase chain reaction, and immunofluorescence analyses.

**Results:**

cAT-MSC-secreted TSG-6 ameliorated IBD and regulated colonic expression of pro- and anti-inflammatory cytokines such as tumor necrosis factor-α, interleukin-6, and interleukin-10. To investigate the effect of cAT-MSC-secreted TSG-6 on activated macrophages in vitro, a transwell coculture system was used; TSG-6 released by cAT-MSCs induced a macrophage phenotypic switch from M1 to M2. The cAT-MSC-secreted TSG-6 increased M2 macrophages in the inflamed colon in vivo.

**Conclusions:**

TSG-6 released from cAT-MSCs can alleviate dextran sulfate sodium-induced colitis by inducing a macrophage phenotypic switch to M2 in mice.

**Electronic supplementary material:**

The online version of this article (10.1186/s13287-018-0841-1) contains supplementary material, which is available to authorized users.

## Background

Inflammatory bowel disease (IBD) is an intractable autoimmune disease that leads to abdominal pain, diarrhea, fever, or other symptoms that may be caused by chronic inflammation of the digestive system. Depending on the pattern and site of inflammation, IBD can be categorized as either Crohn’s disease or ulcerative colitis [1]. Although the exact underlying pathogenesis of IBD is unknown, it is thought to be associated with genetic and environmental factors and gut flora [2, 3]. In addition, the disease occurs naturally in dogs by a similar pathogenesis, and data from therapeutic trials for canine IBD may be excellent references for human IBD [4]. Although IBD leads to a decreased quality of life in both humans and dogs, no effective treatments for IBD have been developed.

Macrophages are important immune cells related to inflammatory diseases and release inflammatory cytokines which are associated with acquired immune cells, such as lymphocytes [5]. In inflamed tissues, macrophages can be classified into two subtypes: M1 and M2 macrophages [6, 7]. M1 macrophages induce inflammatory responses by secreting cytokines such as tumor necrosis factor (TNF)-α, interleukin (IL)-1β, and IL-6, whereas M2 macrophages exert anti-inflammatory responses by releasing anti-inflammatory cytokines such as IL-10 [8]. According to several recent studies, M1 macrophages are present predominantly in inflamed tissues of inflammatory disease model animals; however, the percentage of M2 macrophages increased markedly when the model animals recovered.

Mesenchymal stem cells (MSCs) have been suggested as a promising tool for treating various inflammatory diseases, including pancreatitis, peritonitis, rheumatoid arthritis, and atopic dermatitis, as well as IBD [9–13]. In addition, the mechanisms underlying the therapeutic effects have been investigated and several studies have shown that MSCs exert anti-inflammatory effects through secretory factors [14]. TNF-α-induced gene/protein 6 (TSG-6) is a well-known secretory factor responsible for immunomodulation, and several recent studies have shown that it plays important roles in reducing inflammatory responses in lung injury, corneal injury, skin wound, peritonitis, pancreatitis, and IBD [15–20]. Moreover, MSCs derived from dogs, cats, or horses have been shown to have immunomodulatory effects on activated immune cells and cell-based therapy using MSCs is a potential treatment for intractable inflammatory diseases in veterinary medicine [21–23].

In canine medicine, however, few studies have characterized secretory factors from canine MSCs, and cross-talk mechanisms between canine MSCs and immune cells are not well understood. Evaluating the efficacy of canine MSCs and accumulating therapeutic results from canine medicine would be useful in human medicine as well as veterinary medicine, particularly for intractable inflammatory diseases such as IBD. Therefore, in this study, we assessed the anti-inflammatory effects and mechanisms of canine adipose tissue-derived (cAT)-MSCs in a dextran sulfate sodium (DSS)-induced colitis model.

## Methods

### Isolation and characterization of cAT-MSCs

Canine adipose tissues were collected using a protocol approved by the Institutional Animal Care and Use Committee (IACUC) of Seoul National University (SNU; protocol no. SNU-170724-6). MSCs were isolated from the tissues and cultured as described in Additional file 1: Supplementary materials. Cells were characterized for the expression of several stem cell markers by flow cytometry before they were used in the experiments (Additional file 2: Figure S1). Additionally, the differentiation ability of the cells was confirmed (Additional file 3: Figure S2), and isolated cAT-MSCs at passage 3–4 were used in the following experiments.

### Small interfering RNA (siRNA) transfection of cAT-MSCs

When cAT-MSCs reached approximately 70% confluence they were transfected with TSG-6 siRNA or control siRNA (sc-39,819 and sc-37,007, respectively; Santa Cruz Biotechnology, Santa Cruz, CA, USA) for 24 h using Lipofectamine RNAiMAX (Invitrogen, Carlsbad, CA, USA) according to the manufacturer’s instructions. TSG-6 knockdown was confirmed by quantitative reverse transcription polymerase chain reaction (qRT-PCR) (Additional file 4: Figure S3). cAT-MSCs transfected with siRNA were used for further experiments immediately after the transfection protocol was completed.

### Animal experiments

Male C57BL/6 J mice aged 6 weeks were purchased from Nara Biotech (Seoul, Korea) and housed under controlled temperature, humidity, and light cycle conditions. Only male mice were used in this study to simplify interpretation of the results by avoiding the effects of gender-related differences. All experimental procedures involving animals were approved by the IACUC of SNU (protocol no. SNU-170804-2), and the protocols were performed in accordance with approved guidelines (*n* = 4 for the naive group, *n* = 6 for the other groups). Colitis was induced by ad libitum administration of 3% DSS (36–50 kDa; MP Biomedical, Solon, OH, USA) in the drinking water from day 0 to day 7, whereas mice receiving normal drinking water were used as the naive group. On day 1 the following experiments were performed: 2 × 10^6^ cAT-MSCs transfected with TSG-6 siRNA in 200 μL phosphate-buffered saline (PBS); 2 × 10^6^ cAT-MSCs transfected with scrambled siRNA control in 200 μL PBS; 2 × 10^6^ control cAT-MSCs in 200 μL PBS; or the identical volume of PBS was injected intraperitoneally into the colitis mice. The body weight of each mouse was assessed every 24 h. The mice were sacrificed on day 10 and colon tissues were collected for further processing.

### Evaluating colitis severity

The disease activity index was determined by scoring the body weight loss (grades 0–4: 0, none; 1, < 5% loss of the initial body weight; 2, 5–10% loss of the initial body weight; 3, 10–20% loss of the initial body weight; 4, > 20% loss of the initial body weight), stool consistency (grades 0–2: 0, none; 1, mild to moderate diarrhea; 2, severe diarrhea), rectal bleeding (grades 0–2: 0, none; 1, mild to moderate bleeding; 2, severe bleeding), and general activity (grades 0–2: 0, normal; 1, mildly to moderately depressed; 2, severely depressed).

### Histological analysis

Colon tissues were fixed in 10% formaldehyde for 48 h, embedded in paraffin, and cut into 4-μm sections. The sections were stained with hematoxylin and eosin. A total of 20 fields per group was selected randomly and histological examinations were performed in a blinded manner. The severity of symptoms was calculated by scoring the extent of bowel wall thickening (grades 0–3: 0, none; 1, mucosa; 2, mucosa and submucosa; 3, transmural), damage to the crypt (grades 0–3: 0, none; 1, loss of goblet cells; 2, only surface epithelium intact; 3, loss of entire crypt and epithelium), and infiltration of inflammatory cells (grades 0–2: 0, none; 1, mild to moderate; 2, severe).

### Enzyme-linked immunosorbent assay (ELISA)

Total proteins were extracted from the colon tissue using PRO-PREP Protein Extraction Solution (Intron Biotechnology, Seongnam, Korea) according to the manufacturer’s instructions and stored at −80 °C until use. The concentrations of TNF-α, IL-6, and IL-10 were measured using a commercial ELISA kit (all from eBiosciences, San Diego, CA, USA) according to the manufacturer’s instructions.

### Obtaining canine peripheral blood mononuclear cell (cPBMC)-derived macrophages

Canine macrophages were obtained from the peripheral blood as previously described [24]. Briefly, the blood of healthy canine donors was obtained from the SNU Veterinary Medical Teaching Hospital and PBMCs were isolated using Ficoll-Paque PLUS (GE Healthcare Life Sciences, Little Chalfont, UK). cPBMCs were resuspended in Roswell Park Memorial Institute (RPMI)-1640 medium (PAN Biotech, Aidenbach, Germany) containing 20% fetal bovine serum (FBS; PAN Biotech) and 20% macrophage colony-stimulating factor medium obtained from the supernatant of L929 immortalized cells. The cPBMCs were plated at 2 × 10^6^ cells/well in 24-well plates and incubated at 37 °C in a humidified atmosphere of 5% CO_2_. After 24 h, the wells were washed to remove nonadherent cells. The remaining adherent cells were incubated with fresh medium for 5 days for differentiation into macrophages.

### Coculture of cPBMC-derived macrophages with cAT-MSCs

After cPBMC-derived macrophages were stimulated with 200 ng/ml lipopolysaccharide (LPS; Sigma-Aldrich, St. Louis, MO, USA) for 24 h, the LPS-stimulated macrophages were plated at a density of 2 × 10^5^ cells per well in 24-well plates. Subsequently, 2 × 10^4^ cAT-MSCs, control siRNA-cAT-MSCs, or TSG-6 siRNA-cAT-MSCs were seeded onto 0.4-μm pore-sized Transwell inserts (SPL Life Science, Pocheon, Korea) and incubated for 48 h. The macrophages were harvested for further experiments.

### RNA extraction, cDNA synthesis, and qRT-PCR

Total RNA was extracted from homogenized colon tissue or cPBMC-derived macrophages using the Easy-BLUE Total RNA Extraction kit (Intron Biotechnology) according to the manufacturer’s instructions. cDNA was synthesized using LaboPass M-MuLV Reverse Transcriptase (Cosmo Genetech, Seoul, Korea) and the samples were analyzed using 10 μL AMPIGENE qPCR Green Mix Hi-ROX with SYBR Green dye (Enzo Life Sciences, Farmingdale, NY, USA) and 400 nM forward and reverse primers (Cosmo Genetech). Expression levels of the target genes were normalized to that of glyceraldehyde 3-phosphate dehydrogenase (GAPDH). Primer sequences used in the present study are listed in Additional file 5: Table S1.

### Flow cytometric analysis

Flow cytometry was conducted using a FACSAria II system (BD Biosciences, Franklin Lakes, NJ, USA) and analyzed using FlowJo software (Tree Star, Ashland, OR, USA). To characterize MSCs derived from canine adipose tissues, the cells were harvested and resuspended in PBS. Subsequently, the cells were stained with fluorescein isothiocyanate (FITC)-, phycoerythrin (PE)-, or allophycocyanin (APC)-conjugated antibodies against the following proteins: CD29-FITC, CD34-PE, and CD73-PE (BD Biosciences); and CD44-FITC, CD45-FITC, and CD90-APC (eBiosciences). To evaluate M2 macrophage polarization, PBMC-derived macrophages cocultured with cAT-MSCs were detached and resuspended in PBS. Next, the macrophages were stained with PE-conjugated CD11b (Abcam, Cambridge, UK) and FITC-conjugated CD206 (Santa Cruz Biotechnology).

### Immunofluorescence analysis

Paraffin-embedded colon tissue sections were cut at a thickness of 4 μm. Sections were deparaffinized in xylene and rehydrated sequentially in 100%, 95%, and 80% ethanol solutions, and antigen retrieval was carried out using 10 mM citrate buffer (Sigma-Aldrich). After the sections were washed, they were blocked with blocking buffer containing 5% bovine serum albumin (BSA) and 0.3% Triton X-100 (both from Sigma-Aldrich) for 1 h. The sections were incubated overnight at 4 °C with antibodies against F4/80 (1:250) or FITC-conjugated CD206 (1:250; both from Santa Cruz Biotechnology). After three washes, the slides incubated with F4/80 antibody were incubated with PE-conjugated secondary antibody (1:500; Santa Cruz Biotechnology) for 1 h at 20 °C in the dark. The colon sections stained with antibody against either F4/80 or CD206 were washed three times and mounted in Vectashield mounting medium containing 4′,6-diamidino-2-phenylindole (DAPI; Vector Laboratories, Burlingame, CA, USA). The slides were visualized with a confocal laser scanning microscope (LSM710; Carl Zeiss, Jena, Germany), and immunoreactive cells were counted in 20 random fields per group.

### Annexin-V and propidium iodide (PI) staining

Colon tissue slides were stained with FITC-conjugated annexin-V and PI using the Annexin V-FITC apoptosis detection kit plus (Enzo Life Sciences) according to the manufacturer’s instructions. The slides were observed using an EVOS FL microscope (Life Technologies, Carlsbad, CA, USA). Apoptotic cells identified as FITC-positive cells were counted in 20 random fields per group.

### Generation of the GAPDH standard curve

Standard curves for evaluating the migratory ability of intraperitoneally injected canine MSCs were generated by administering serial dilutions of cAT-MSCs to mouse organs as described previously [25]. Briefly, 2 × 10^2^, 2 × 10^3^, 2 × 10^4^, or 2 × 10^5^ cAT-MSCs were added to whole mouse organs prior to homogenization. Total RNA was extracted from the samples using the Easy-BLUE Total RNA Extraction kit (Intron Biotechnology), and cDNA was synthesized (LaboPass M-MuLV Reverse Transcriptase; Cosmo Genetech) using 1 μg of RNA. Next, qRT-PCR using canine-specific mitochondrial cytochrome b primers (forward primer, 5′-CCT TAC TAG GAG TAT GCT TG-3′; reverse primer, 5′-TGG GTG ACT GAT GAA AAA G-3′) was performed to generate the standard curves. The curves were corrected by performing parallel qRT-PCR with primers for universal eukaryotic 18S ribosomal RNA (forward primer, 5′-GCT ACT ACC GAT TGG ATG GTT TAG-3′; reverse primer, 5′-CTA CGG AAA CCT TGT TAC GAC TTT-3′).

### Statistical analysis

Data are shown as the mean ± standard deviation. Mean values among different groups were compared by one-way analysis of variance using the GraphPad Prism v.6.01 software (GraphPad, Inc., La Jolla, CA, USA). A *P* value < 0.05 was considered statistically significant.

## Results

### Intraperitoneally administered cAT-MSC-secreted TSG-6 plays a crucial role in ameliorating IBD

**Fig. 1:**
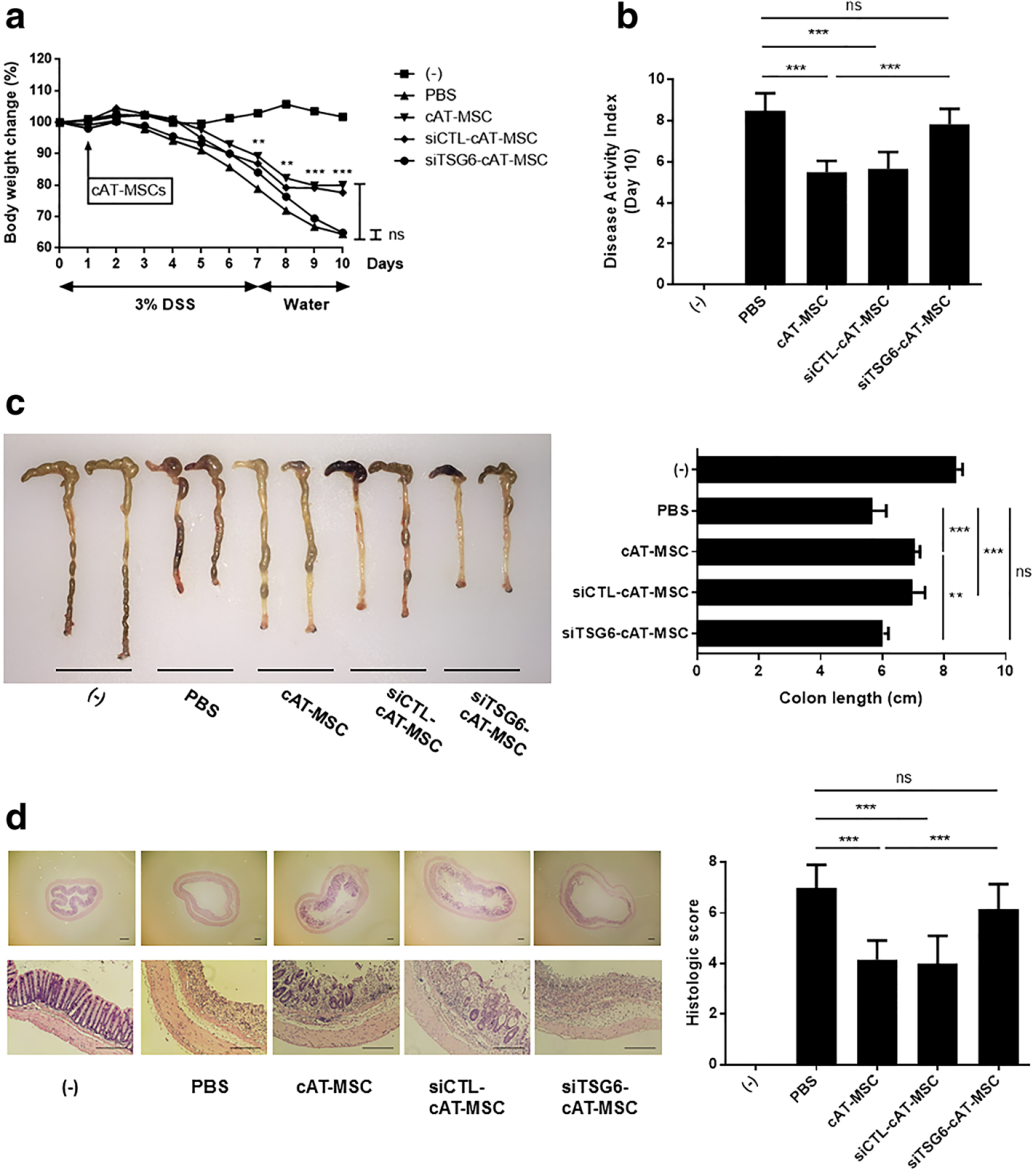
Intraperitoneally infused cAT-MSC-secreted TSG-6 plays an essential role in alleviating IBD. Dextran sulfate sodium (DSS)-induced colitis mice were administered with canine adipose tissue-derived mesenchymal stem cells (cAT-MSCs) transfected with tumor necrosis factor-α-induced gene/protein-6 (TSG-6) small interfering (si)RNA (siTSG6-cAT-MSC), cAT-MSCs transfected with scrambled siRNA (siCTL-cAT-MSC), naive cAT-MSCs, or phosphate-buffered saline (PBS; vehicle control) on day 1. **a** Body weight was measured every day and expressed in terms of the relative change from the weight measured on day 0. Mice were sacrificed on day 10 and **b** Disease Activity Index (DAI) and **c** colon length were assessed. **d** Representative hematoxylin and eosin staining of the colon tissue sections and their histological scores are shown. Scale bars = 100 μm. Four to six mice per group were used. Results are shown as the mean ± standard deviation. **P < 0.01, ***P < 0.001. ns, not significant

We have previously shown the therapeutic effects of TSG-6 released from human AT-MSCs against colitis [19]. In this study, we first investigated whether cAT-MSC-secreted TSG-6 exerted anti-inflammatory effects in DSS-induced colitis mice. Intraperitoneally infused cAT-MSCs significantly reduced body weight loss compared with mice injected with PBS from day 7 (Fig. 1a). On day 10, the disease activity index of colitis mice treated with cAT-MSCs was significantly improved compared with mice treated with PBS (Fig. 1b). On day 10, mice were sacrificed to evaluate the length and histology of the colon. The shortening of colon length was significantly improved in the cAT-MSC-treated group compared with the PBS-treated group (Fig. 1c). Upon histological examination, severe submucosal or transmural thickening, destruction of the entire epithelium, and severe inflammatory cell infiltration were observed in DSS-induced colitis mouse colons. In colon sections from mice treated with cAT-MSCs, the extent of bowel wall thickening, crypt damage, and infiltration of inflammatory cells were improved compared with PBS-treated mice (Fig. 1d). However, colitis mice administered with cAT-MSCs transfected with TSG-6 siRNA did not show improvements in body weight loss, disease activity index, colon length, or histologic scores compared with colitis mice injected with PBS (Fig. 1a–d).

### cAT-MSC-secreted TSG-6 reduced inflammatory response and apoptosis in the colon

**Fig. 2:**
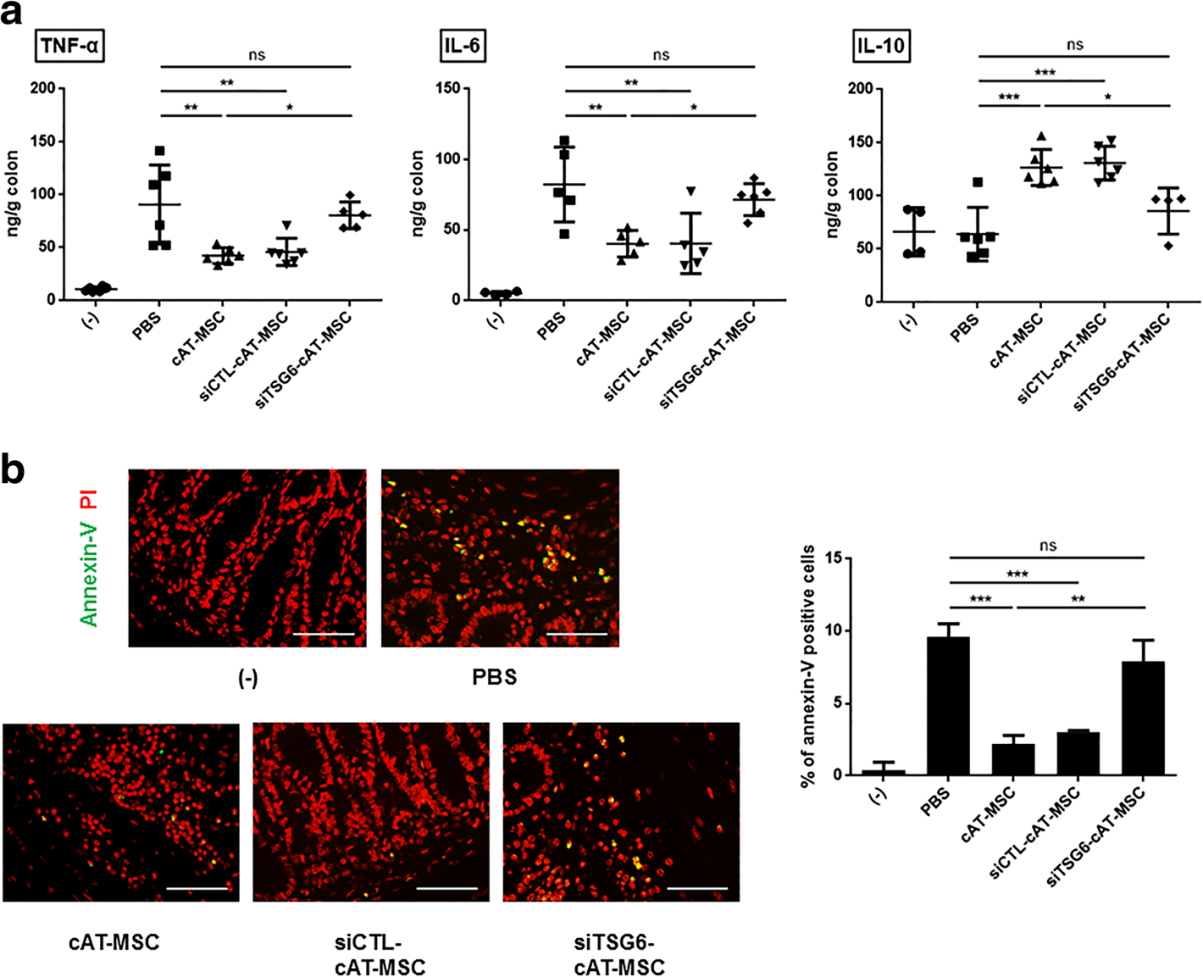
TSG-6 secreted by canine adipose tissue-derived mesenchymal stem cells (cAT-MSCs) inhibits inflammatory response and apoptosis in the colon. **a** Levels of tumor necrosis factor (TNF)-α, interleukin (IL)-6, and IL-10 in colons were assessed by ELISA. **b** Representative immunofluorescence staining of colon tissue sections using annexin-V antibody or propidium iodide (PI), and the percentage of the annexin-V-positive cells are shown. Four to six mice per group were used. Results are shown as the mean ± standard deviation. *P < 0.05, **P < 0.01, ***P < 0.001. ns, not significant; PBS, phosphate-buffered saline (vehicle control); siCTL-cAT-MSC, cAT-MSCs transfected with scrambled small interfering RNA; siTSG6-cAT-MSC, cAT-MSCs transfected with TSG-6 tumor necrosis factor-α-induced gene/protein-6 small interfering RNA

We next evaluated the effect of cAT-MSCs on the modulation of inflammatory cytokines associated with IBD or DSS-induced colitis. Production of TNF-α and IL-6 was considerably increased in the colon of DSS-treated mice, whereas that of IL-10 was slightly decreased (Fig. 2a). Treatment with cAT-MSCs not only significantly decreased TNF-α and IL-6, but also significantly increased IL-10 (Fig. 2a). However, siRNA-induced downregulation of TSG-6 significantly reduced the anti-inflammatory abilities of cAT-MSCs to modulate TNF-α, IL-6, and IL-10 in the colon (Fig. 2a).

In addition, we examined apoptosis that could be induced by TNF-α in colon sections. Annexin-V-positive cells were increased markedly in the colons of DSS-induced mice (Fig. 2b). Administration of cAT-MSCs led to a significant decrease in annexin-V-positive cells in the colon sections compared with the PBS-treated groups, which was abolished by knockdown of TSG-6 with siRNA transfection (Fig. 2b).

### Intraperitoneally infused cAT-MSCs did not migrate to the inflamed colon

**Fig. 3:**
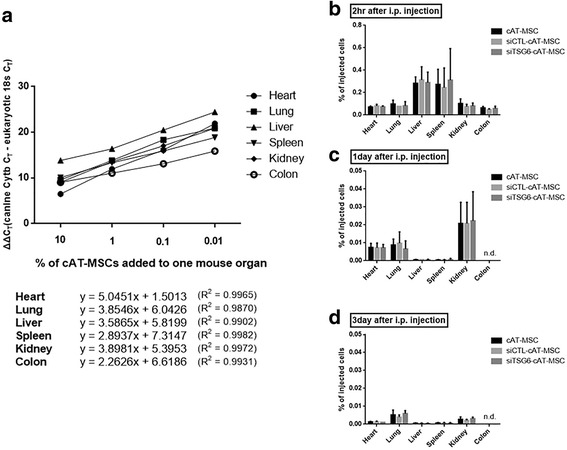
Intraperitoneally administered canine adipose tissue-derived mesenchymal stem cells (cAT-MSCs) do not migrate into the colon. **a** Standard curves for evaluating the migratory ability of intraperitoneally (i.p.) injected cells were generated by administering serial dilutions of cAT-MSCs to the mouse heart, lung, liver, spleen, kidney, and colon. **b–d** The percentage of infused cAT-MSCs in the organs was evaluated **b** at 2 h, **c** at 1 day, and **d** at 3 days after cell administration. Results are presented as the mean ± standard deviation of the data obtained in three independent experiments. nd, not detected; siCTL-cAT-MSC, cAT-MSCs transfected with scrambled small interfering RNA; siTSG6-cAT-MSC, cAT-MSCs transfected with TSG-6 tumor necrosis factor-α-induced gene/protein-6 small interfering RNA

Next, we tracked and quantified intraperitoneally injected cAT-MSCs (2 × 10^6^ cells) by constructing standard curves by qRT-PCR (Fig. 3a). After 2 h of cAT-MSC injection, approximately 0.07%, 0.11%, 0.26%, 0.25%, 0.08%, and 0.06% of the cells were detected in the heart, lung, liver, spleen, kidney, and colon of DSS-induced colitis mice, respectively (Fig. 3b). At days 1 and 3 after cAT-MSC administration, these percentages were lower than they were at 2 h after cell infusion (Fig. 3c, d). Furthermore, infused cAT-MSCs were not detected in inflamed colons at days 1 and 3 (Fig. 3c, d).

### TSG-6 produced by cAT-MSCs induced phenotypic switching from M1 to M2 macrophages in vitro

**Fig. 4:**
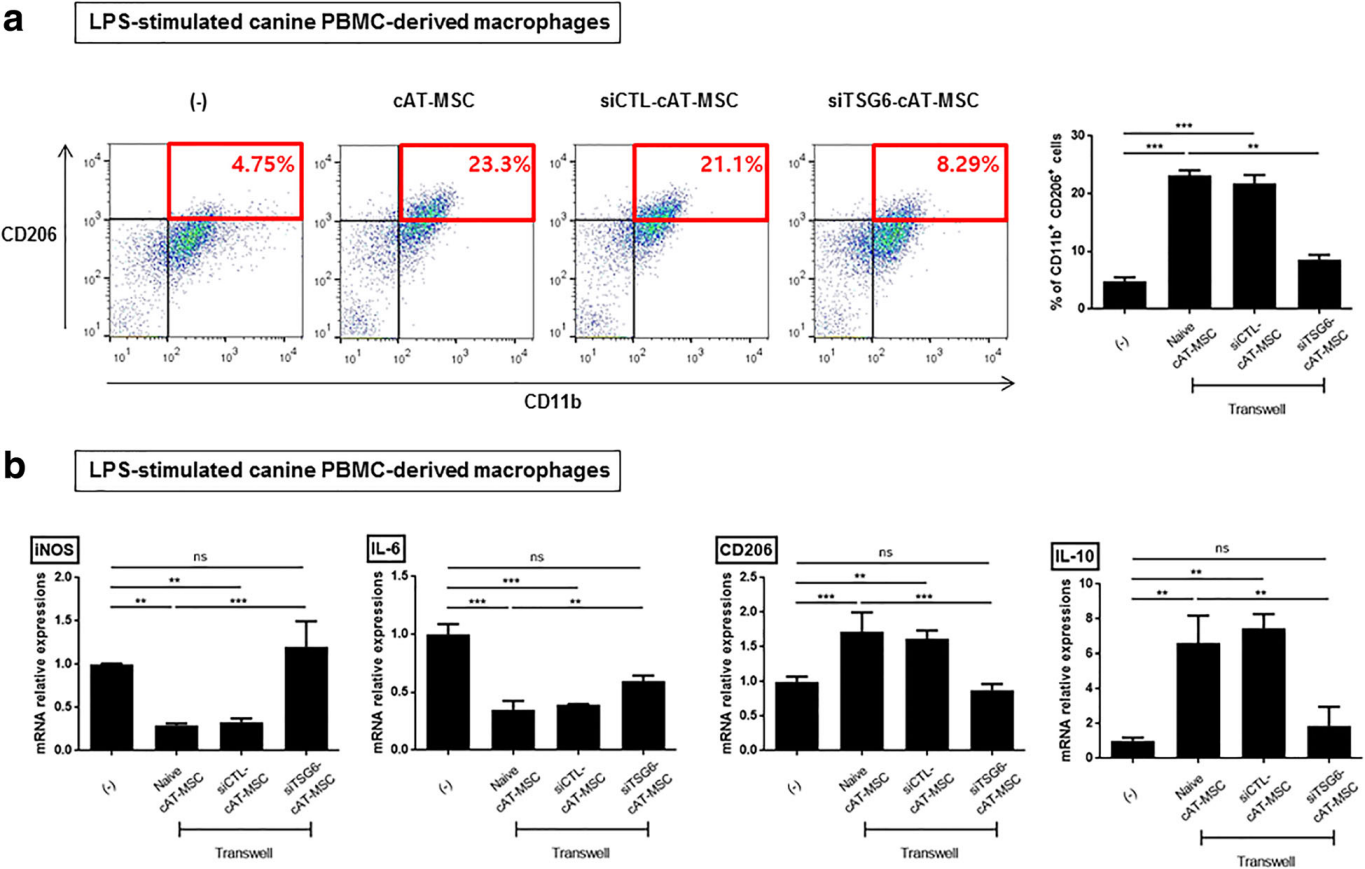
Canine adipose tissue-derived mesenchymal stem cell (cAT-MSC)-secreted TSG-6 induces macrophage phenotypic switching from M1 to M2 *in vitro*. Lipopolysaccharide (LPS)-stimulated canine peripheral blood mononuclear cell (PBMC)-derived macrophages were cocultured in a transwell system with cAT-MSCs transfected with tumor necrosis factor-α-induced gene/protein-6 (TSG-6) small interfering (si)RNA (siTSG6-cAT-MSC), cAT-MSCs transfected with scrambled small interfering RNA (siCTL-cAT-MSC), or naive cAT-MSCs for 48 h. **a** M2 macrophage population was determined by measuring CD11b and CD206 double-positive cells by flow cytometry. **b** Inducible nitric oxide synthase (iNOS), interleukin (IL)-6, CD206, and IL-10 mRNA expression levels in the macrophages were evaluated. Results are presented as the mean ± standard deviation of three independent experiments. **P < 0.01, ***P < 0.001. ns, not significant

Given that cytokines such as TNF-α, IL-6, and IL-10 modulated in the above study are largely derived from macrophages, we further investigated whether cAT-MSCs could switch the macrophage phenotype from M1 to M2. Macrophages derived from cPBMCs were stimulated with LPS (200 ng/ml) for 24 h to induce the M1 phenotype. Next, macrophages were cocultured with cAT-MSCs in a transwell system for 48 h. The proportion of CD11b^+^ cells expressing CD206, a well-known M2 marker, was significantly increased in the cAT-MSC group compared with the control group (Fig. 4a). In addition, cAT-MSCs transiently transfected with TSG-6 siRNA were cocultured with LPS-stimulated cPBMC-derived macrophages to determine whether TSG-6 mediates macrophage polarization. Interestingly, the phenotypic switching effect was significantly decreased in cAT-MSCs transfected with TSG-6 siRNA compared with cAT-MSCs transfected with control siRNA or naive cAT-MSCs (Fig. 4a). Furthermore, a reduction in mRNA expression of inducible nitric oxide synthase (iNOS) and IL-6 was observed in LPS-stimulated cPBMC-derived macrophages cocultured with cAT-MSCs compared with those incubated alone, which was abrogated by coculture with cAT-MSCs transfected with TSG-6 siRNA (Fig. 4b). Additionally, the CD206 and IL-10 mRNA expression levels of LPS-stimulated cPBMC-derived macrophages were increased in the cAT-MSC group compared with the control group, which were restored in the TSG-6 siRNA-transfected cAT-MSC group (Fig. 4b).

### cAT-MSC-secreted TSG-6 increased M2 macrophages in the inflamed colon

**Fig. 5:**
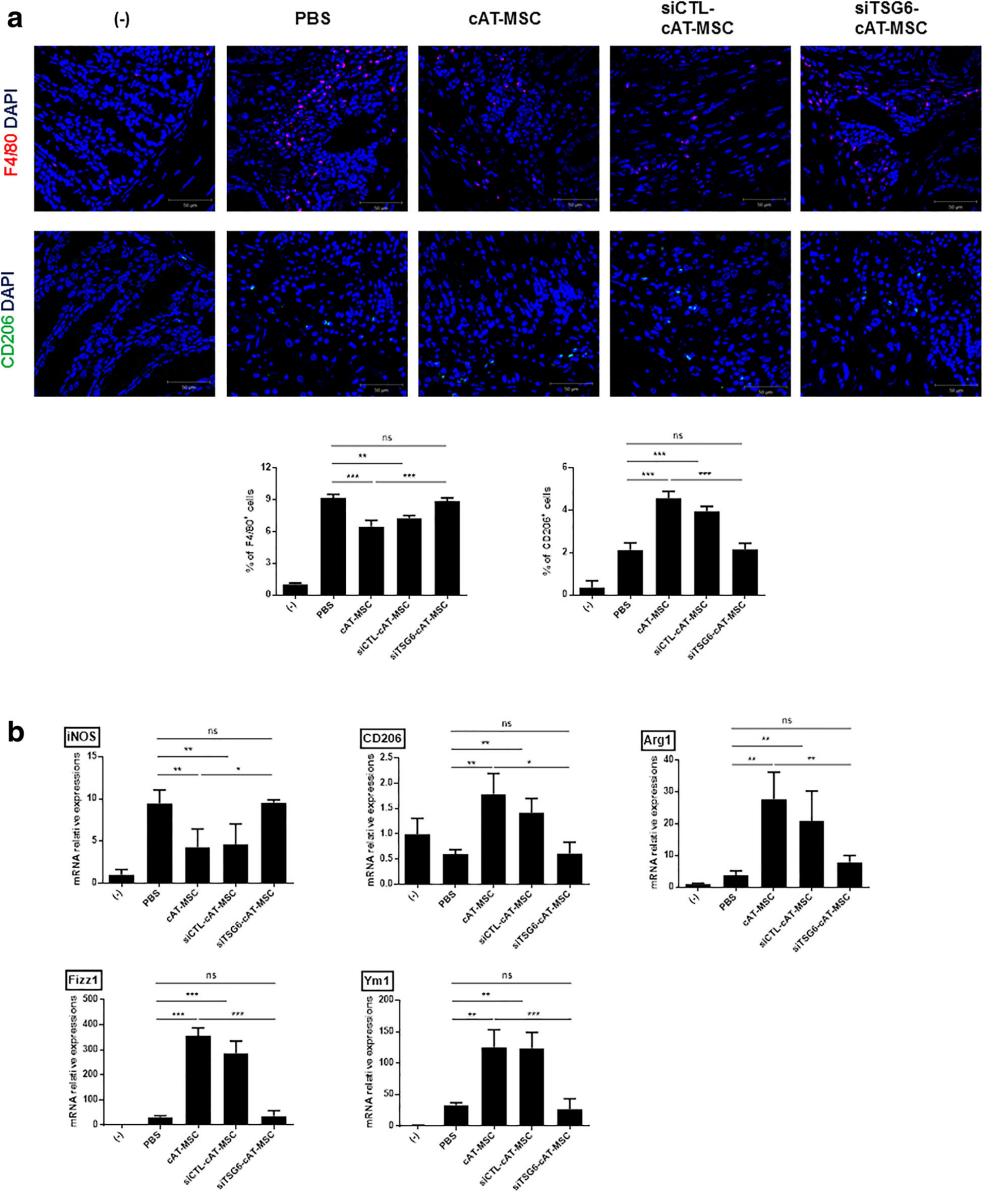
TSG-6 secreted by canine adipose tissue-derived mesenchymal stem cells (cAT-MSCs) induces M2 macrophage polarization in the inflamed colon. **a** Representative immunofluorescence staining of colon tissue sections using F4/80-specific or CD206-specific antibodies, and the percentages of F4/80-positive or CD206-positive cells are shown. Scale bars = 50 μm. **b** The gene expression levels of inducible nitric oxide synthase (iNOS), CD206, Arg1, Fizz1, and Ym1 in the colon samples of mice were evaluated. Four to six mice per group were used. Results are presented as the mean ± standard deviation. *P < 0.05, **P < 0.01, ***P < 0.001. ns, not significant; PBS, phosphate-buffered saline (vehicle control); siCTL-cAT-MSC, cAT-MSCs transfected with scrambled small interfering RNA; siTSG6-cAT-MSC, cAT-MSCs transfected with TSG-6 tumor necrosis factor-α-induced gene/protein-6 small interfering RNA

We next assessed the expression level of M2 macrophages in inflamed colons. Quantitative analysis of macrophages detected in colon tissue sections by immunofluorescence examination showed that the percentage of F4/80^+^ total macrophages was decreased significantly, whereas that of CD206^+^ M2 macrophages was increased significantly in the cAT-MSC group compared with the PBS group (Fig. 5a). Furthermore, the mRNA expression level of iNOS was significantly decreased and that of the M2 markers CD206, Arg1, Fizz1, and Ym1 were markedly increased in colon tissues of DSS-induced colitis mice infused with cAT-MSCs compared with PBS-treated mice (Fig. 5b). However, the M2 polarization effect of cAT-MSCs in colon tissue was abrogated when TSG-6 was inhibited (Fig. 5).

## Discussion

Numerous studies have shown that MSC administration may be an important treatment option for IBD [26–31]. Although they are not fully understood, the mechanisms underlying the anti-inflammatory effects of MSC have been described previously [32–34]. It is well known that the anti-inflammatory ability and mechanisms of MSCs vary depending on their source [35]. Moreover, inflammatory cytokines released by activated immune cells are important triggers for MSCs to exert immunomodulatory properties, indicating that the disease-specific inflammatory microenvironment is crucial for the therapeutic effects of administered MSCs [36]. In this study, we first demonstrated the anti-inflammatory effects of cAT-MSCs and determined the underlying mechanisms in a DSS-induced colitis mouse model.

Recent studies reported that MSCs reduce inflammation through soluble factors such as indoleamine 2,3-dioxygenase, transforming growth factor-β, prostaglandin E2 (PGE2), hepatocyte growth factor, and TSG-6 [12, 32, 33, 37, 38]. Among these, TSG-6 has been shown to be pivotal for the immunomodulatory effects of MSCs in several inflammatory disease models such as corneal inflammation, wound injury, acute lung injury, peritonitis, and pancreatitis [10, 16, 17, 20, 39]. We also previously demonstrated that MSCs derived from human adipose tissue exert therapeutic effects against DSS-induced colitis by secreting TSG-6 [19]. Here, we showed that TSG-6 secreted from intraperitoneally infused cAT-MSCs may ameliorate the symptoms of DSS-induced colitis and that weight loss and disease activity indices were reduced. In addition, by evaluating the length of the colon and assessing the histologic scores of the colon tissue sections, we demonstrated the therapeutic effects of cAT-MSC-secreted TSG-6 against DSS-induced colitis mice. Moreover, TSG-6 released from cAT-MSCs played an important role in modulating inflammatory cytokines such as TNF-α, IL-6, and IL-10 in the colon; reduced TNF-α secretion led to a significant decrease in annexin V-positive apoptotic cells in colon tissue sections.

In the present study, we administered cAT-MSCs into an immunocompetent mouse model of IBD. We focused on alterations in mouse immune cells after treatment with canine MSCs. Moreover, MSCs are immunoprivileged, partly because of the low expression of major histocompatibility complex class II molecules [40]. Similar approaches involving the injection of xenogeneic MSCs into immunocompetent mouse models have been used by several groups, and cross-species-induced immunological responses have not been reported [17, 21, 39, 41]. Additionally, in this study, none of the mice injected with cAT-MSCs showed any side effects or died until sacrifice. In addition, Wang et al. reported that intraperitoneal infusion of MSCs showed better amelioration of DSS-induced colitis compared with local anal injection, suggesting that systemic immunomodulation rather than reducing local inflammation is required for effective therapy [42]. Therefore, we determined that the optimal conditions were 2 × 10^6^ cAT-MSCs infused intraperitoneally.

We also evaluated the distribution of intraperitoneally administered cAT-MSCs using qRT-PCR, which is known to have relatively higher sensitivity and specificity compared to fluorescence-mediated cell tracking. Two hours after cAT-MSC infusion less than 1% of the infused cells were detected in the heart, lung, liver, spleen, kidney, and colon tissues. At 1 and 3 days after cAT-MSC administration, the percentages of cells detected in the tissues were less than 0.5%. Furthermore, infused cAT-MSCs were not detected in colon tissues, despite inflammatory responses observed at days 1 and 3. Similar results were obtained when cAT-MSCs transiently transfected with control or TSG-6 siRNA were administered. These results are consistent with those of our previous study, indicating that intraperitoneally injected cAT-MSCs formed aggregates in the peritoneal cavity and alleviated DSS-induced colitis at sites distant from the colon through soluble factors, such as TSG-6 [19].

Considering that inflammatory cytokines modulated in the inflamed colon treated with cAT-MSCs were principally derived from macrophages, we carried out in vitro experiments to evaluate whether TSG-6 secreted from cAT-MSCs could switch the macrophage phenotype from M1 to M2. LPS-stimulated cPBMC-derived macrophages exhibit a conventional M1 type pattern and were cocultured with cAT-MSCs transfected with siRNA or with naive cAT-MSCs in a transwell system. CD206-expressing M2 macrophages were increased markedly in the cAT-MSC group but were inhibited in the siTSG6-cAT-MSC group. Moreover, TSG-6 secreted from cAT-MSCs contributed to the decrease in the expression levels of M1 markers such as iNOS and IL-6.

Next, we evaluated M2 macrophages in colon tissue sections of DSS-induced colitis mice treated with cAT-MSCs to analyze the macrophage polarization ability of TSG-6 released by cAT-MSCs in vivo. Recent studies suggested that MSCs have the capacity to induce phenotypic alterations in macrophages in acute kidney injury, spinal cord injury, and skin wound animal models [43–45]. Consistent with these reports, the expression levels of M2 markers in the colon tissue were increased in the siCTL-cAT-MSC-treated group and naive cAT-MSC-treated group compared with the PBS-treated group. However, the siTSG6-cAT-MSC-treated group showed no significant changes in the expression levels of these markers compared with the PBS group. Taken together, we demonstrated that TSG-6 secreted by cAT-MSCs plays an essential role in switching the phenotype of macrophages from M1 to M2 in the inflamed colon.

We could not rule out the possibility that other factors secreted from cAT-MSCs contributed to M2 macrophage polarization in colitis mice. Interestingly, other groups recently showed that PGE2 released by human MSCs induced macrophage phenotypic alteration [46, 47]. Further experiments on other factors secreted from cAT-MSCs, such as PGE2, are required to verify their effects on macrophages in IBD models. However, our findings suggest that TSG-6 released by cAT-MSCs plays an important role in switching the macrophage phenotype from M1 to M2 in vitro and in DSS-induced colitis mice.

## Conclusion

In conclusion, we demonstrated that TSG-6 secreted by cAT-MSCs ameliorated DSS-induced colitis in mice by inducing macrophages to switch to the M2 phenotype. In addition, our findings reveal a possible mechanism underlying the TSG6-induced therapeutic effects in various inflammatory disease models previously published. These results may help in the development of cell-based therapies for treating IBD.

## Additional files

Additional file 1: **Supplementary materials.** Canine adipose tissue-derived mesenchymal stem cells: isolation, culture, and characterization. (PDF 16 kb)

Additional file 2: **Figure S1.** cAT-MSCs have high expression of CD29, CD73, CD44, and CD90, and do not express CD31, CD34, or CD45. (TIFF 3502 kb)

Additional file 3: **Figure S2.** cAT-MSCs have the ability to differentiate into adipocytes (Oil Red O staining), osteocytes (Alizarin Red S staining), and chondrocytes (Alcian Blue staining). Scale bars = 200 μm. (TIFF 9973 kb)

Additional file 4: **Figure S3.** mRNA expression level of TSG-6 of naive cAT-MSCs, cAT-MSCs treated with transfection reagents only (cAT-MSC + transfection reagent), cAT-MSCs transfected with a scrambled siRNA (siCTL-cAT-MSC), or cAT-MSCs transfected with TSG-6 siRNA (siTSG6-cAT-MSC) was determined by real-time RT-PCR. Results are presented as the mean ± standard deviation of three independent experiments. ***P < 0.001. (TIFF 1818 kb)

Additional file 5: **Table S1.** Primers used for this study. (PDF 33 kb)

## Abbreviations

APC: Allophycocyanin
cAT-MSC: Canine adipose tissue-derived mesenchymal stem cell
cPBMC: Canine peripheral blood mononuclear cell
DSS: Dextran sulfate sodium
ELISA: Enzyme-linked immunosorbent assay
FITC: Fluorescein isothiocyanate
GAPDH: Glyceraldehyde 3-phosphate dehydrogenase
IACUC: Institutional Animal Care and Use Committee
IBD: Inflammatory bowel disease
IL: Interleukin
iNOS: Inducible nitric oxide synthase
LPS: Lipopolysaccharide
MSC: Mesenchymal stem cell
PBS: Phosphate-buffered saline
PE: Phycoerythrin
PGE2: Prostaglandin E2
PI: Propidium iodide
qRT-PCR: Quantitative reverse transcription polymerase chain reaction
siRNA: Small interfering ribonucleic acid
SNU: Seoul National University
TNF: Tumor necrosis factor
TSG-6: Tumor necrosis factor-α-induced gene/protein-6
