# Supplementary material for: TSG-6 released from intraperitoneally injected canine adipose tissue-derived mesenchymal stem cells ameliorate inflammatory bowel disease by inducing M2 macrophage switch in mice
Source: Stem Cell Res Ther. 2018 Apr 6;9:91. doi: 10.1186/s13287-018-0841-1 (PMC5889600; doi:10.1186/s13287-018-0841-1)
Supplement: Supplementary file 1 — Supplementary materials. Canine adipose tissue-derived mesenchymal stem cells: isolation, culture, and characterization. (PDF 16 kb) [file 13287_2018_841_MOESM1_ESM.pdf]

Supplementary materials

cAT-MSCs isolation and culturing

Canine adipose tissue was obtained from a healthy dog <1-year-old under a protocol approved by the IACUC of Seoul National University (SNU; protocol no. 170724-6). The tissue was washed three times with PBS (PAN Biotech) containing penicillin and streptomycin, and then cut into small pieces and digested for 1 h at 37°C with collagenase type IA (1 mg/mL; Sigma-Aldrich). Enzymatic activity was inhibited with Dulbecco's Modified Eagle's Medium (DMEM; PAN Biotech) containing 10% fetal bovine serum (FBS; PAN Biotech). After centrifugation at 1200 ×*g* for 5 min, the pellet was filtered through a 100-µm Falcon cell strainer (Fisher Scientific, Waltham, MA, USA) to remove debris and then incubated in DMEM containing 10% FBS at 37°C in a humidified atmosphere of 5% CO_2. After 48 h, the cultures were washed with PBS to remove non-adherent cells and incubated with fresh medium, which was changed every 48 h until cells reached 70–80% confluence, after which they were repeatedly subcultured under standard conditions. Isolated cAT-MSCs were used at passage 3–4 for the following experiments.

Characterization of cAT-MSCs

Isolated cells were characterized for the expression of stem cell markers by flow cytometry using fluorescein isothiocyante (FITC)- or phycoerythrin (PE)-conjugated antibodies against the following proteins: CD29-FITC, CD31-FITC, CD34-PE, and CD73-PE (BD Biosciences) and CD44-FITC, CD45-FITC, and CD90-APC (eBiosciences). Cells were analyzed with a FACSAria II system (BD Biosciences). Cellular differentiation was evaluated using the StemPro Adipogenesis Differentiation, StemPro Osteogenesis Differentiation, and StemPro Chondrogenesis Differentiation kits (all from Gibco/Life Technologies, Carlsbad, CA, USA)

according to the manufacturer’s instructions followed by Oil Red O staining, Alizarin Red staining, and Alcian Blue staining, respectively.
