# Supplementary figures and images for: TSG-6 released from intraperitoneally injected canine adipose tissue-derived mesenchymal stem cells ameliorate inflammatory bowel disease by inducing M2 macrophage switch in mice
Source: Stem Cell Res Ther. 2018 Apr 6;9:91. doi: 10.1186/s13287-018-0841-1 (PMC5889600; doi:10.1186/s13287-018-0841-1)

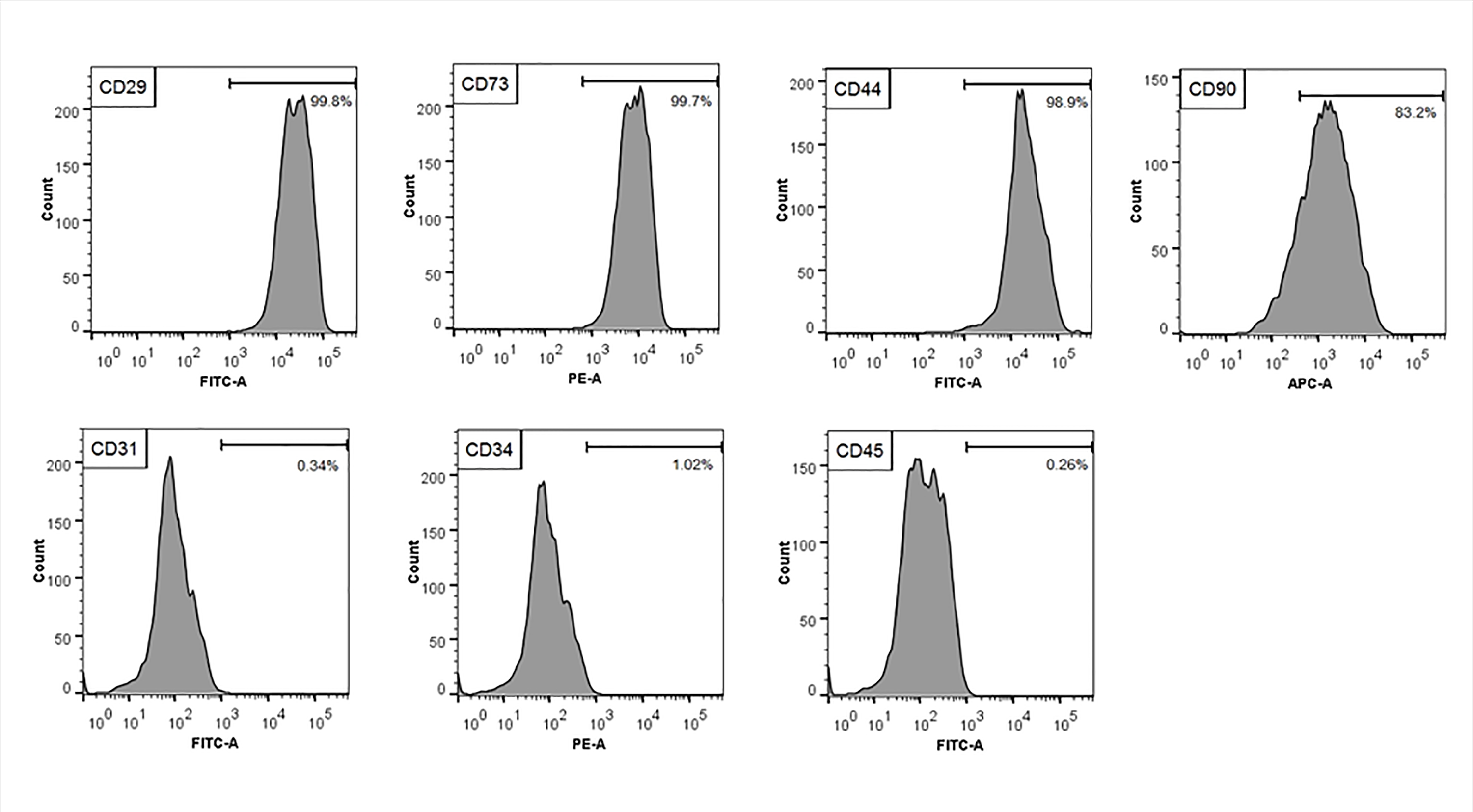

Supplement: Supplementary file 2 — Figure S1. cAT-MSCs have high expression of CD29, CD73, CD44, and CD90, and do not express CD31, CD34, or CD45. (TIFF 3502 kb) [file 13287_2018_841_MOESM2_ESM.tif]

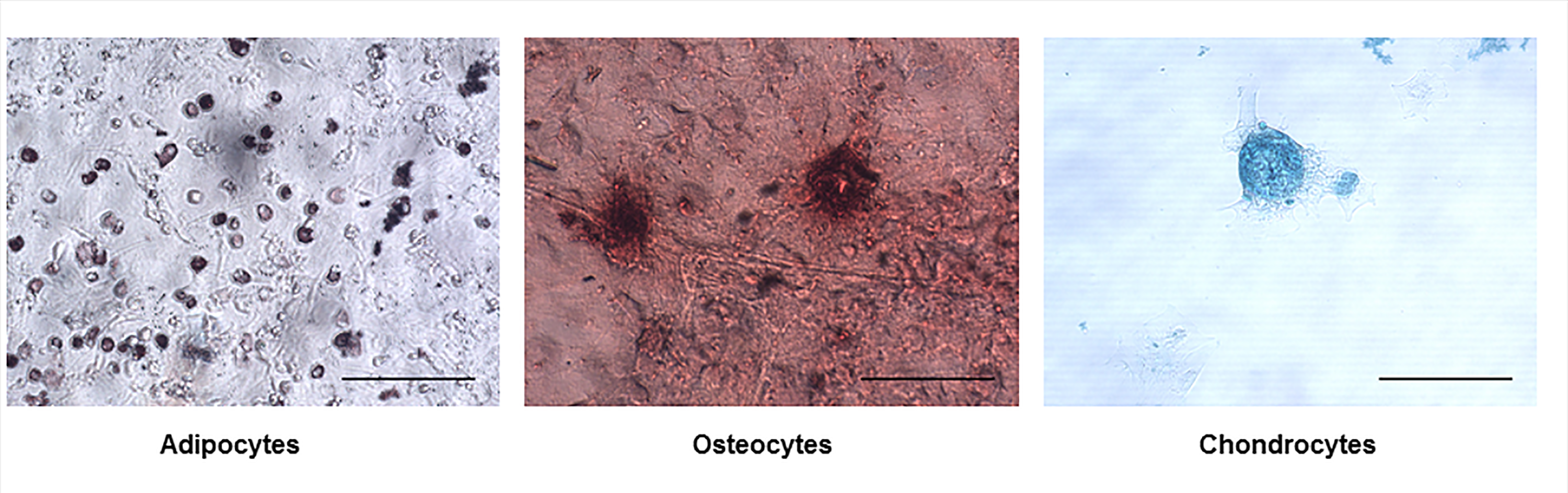

Supplement: Supplementary file 3 — Figure S2. cAT-MSCs have the ability to differentiate into adipocytes (Oil Red O staining), osteocytes (Alizarin Red S staining), and chondrocytes (Alcian Blue staining). Scale bars = 200 μm. (TIFF 9973 kb) [file 13287_2018_841_MOESM3_ESM.tif]

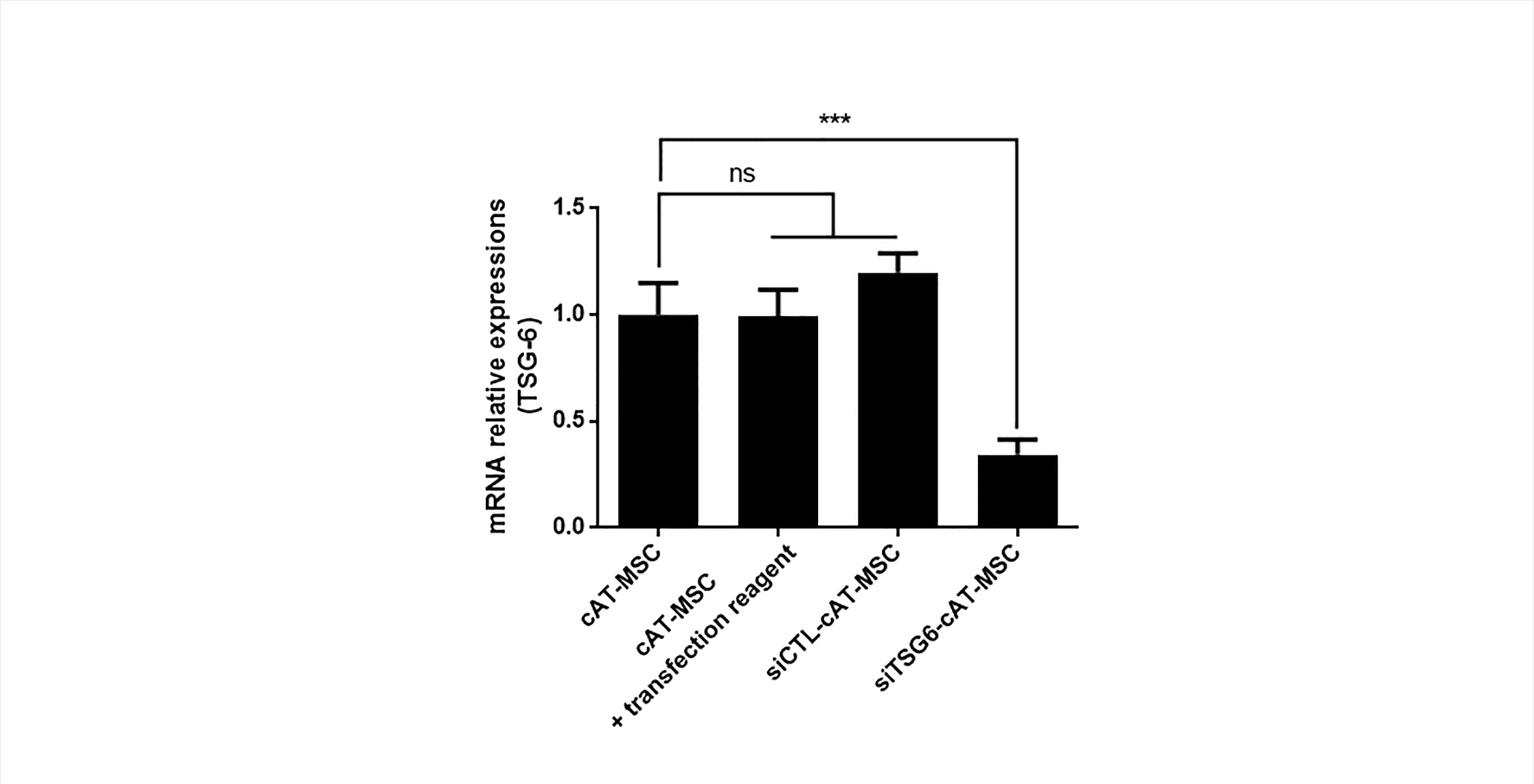

Supplement: Supplementary file 4 — Figure S3. mRNA expression level of TSG-6 of naive cAT-MSCs, cAT-MSCs treated with transfection reagents only (cAT-MSC + transfection reagent), cAT-MSCs transfected with a scrambled siRNA (siCTL-cAT-MSC), or cAT-MSCs transfected with TSG-6 siRNA (siTSG6-cAT-MSC) was determined by real-time RT-PCR. Results are presented as the mean ± standard deviation of three independent experiments. ***P < 0.001. (TIFF 1818 kb) [file 13287_2018_841_MOESM4_ESM.tif]
