# Supplementary material for: TSG-6 released from intraperitoneally injected canine adipose tissue-derived mesenchymal stem cells ameliorate inflammatory bowel disease by inducing M2 macrophage switch in mice
Source: Stem Cell Res Ther. 2018 Apr 6;9:91. doi: 10.1186/s13287-018-0841-1 (PMC5889600; doi:10.1186/s13287-018-0841-1)
Supplement: Supplementary file 5 — Table S1. Primers used for this study. (PDF 33 kb) [file 13287_2018_841_MOESM5_ESM.pdf]

## Supplementary tables

**Table S1.** List for primers for this study

| Gene | Forward (5′-3′) | Reverse (5′-3′) |
| --- | --- | --- |
| **<u>Mouse</u>** | | |
| iNOS | AAAGGAAATAGAAACAACAGGAACC | GCATAAAGTATGTGTCTGCAGATGT |
| IL-6 | AGGCTTAATTACACATGTTCTCTGG | TTATATCCAGTTTGGTAGCATCCAT |
| CD206 | AACGGAATGATTGTGTAGTTCTAGC | TACAGGATCAATAATTTTTGGCATT |
| Arg1 | CAGAAGAATGGAAGAGTCAG | CAGATATGCAGGGAGTCACC |
| Fizz1 | GAATCTATTGTGGAGAAAAAGGTCA | AGCCGTGATACTAGTACAGGAGAAA |
| Ym1 | GTGTACTCACCTGATCTATGCCTTT | CAGGAGAGTTTTTAGCTCAGTGTTC |
| IL-10 | GTGATTTTAATAAGCTCCAAGACCA | GATCATCATGTATGCTTCTATGCAG |
| GAPDH | AGTATGTCGTGGAGTCTACTGGTGT | AGTGAGTTGTCATATTTCTCGTGGT |
| **<u>Canine</u>** | | |
| iNOS | AAATTATGTCCTGTCCCCTTTCTAC | TTTAAGTTGAATCTTTTTCCTGTGG |
| IL-6 | ATGATCCACTTCAAATAGTCTACC | AGATGTAGGTTATTTTCTGCCAGTG |
| CD206 | GGAAATATGTAAACAGGAATGATGC | TCCATCCAAATAAACTTTTTATCCA |
| IL-10 | ATTTCTGCCCTGTGAGAATAAGAG | TGTAGTTGATGAAGATGTCAAGCTA |
| TSG-6 | TCCGTCTTAATAGGAGTGAAAGATG | AGATTTAAAAATTCGCTTTGGATCT |
| GAPDH | TTAACTCTGGCAAAGTGGATATTGT | GAATCATACTGGAACATGTACACCA |
